# Investigation of an *MCM8* gene variant in women with premature ovarian insufficiency

**DOI:** 10.31744/einstein_journal/2024AO0712

**Published:** 2024-10-23

**Authors:** Beatriz Xavier de Camargo Rabello, Laura Alves da Rocha, Caio Parente Barbosa, Bianca Bianco, Denise Maria Christofolini

**Affiliations:** 1 Centro Universitário FMABC Santo André SP Brazil Centro Universitário FMABC, Santo André, SP, Brazil.

**Keywords:** Minichromosome maintenance proteins, Primary ovarian insufficiency, Ovarian diseases

## Abstract

**Introduction:**

The *MCM8* gene is involved in the homologous recombination and repair of double-stranded DNA breaks. It maintains the meiotic process continuously. If the MCM8-9 helicase does not function, the accumulation of DNA breaks can result in cell death. Studies have reported *MCM8* gene suppression with primary ovarian insufficiency. In the present study, a variant of the *MCM8* gene was investigated in women with primary ovarian insufficiency to elucidate the role of *MCM8* in this pathology.

**Objective:**

To evaluate the frequency of the NG_042869.1:g.40270G>A variant of the *MCM8* gene in the study population.

**Methods:**

The *MCM8* gene variant was analyzed via real-time polymerase chain reaction using a hydrolysis probe in DNA samples from women diagnosed with primary ovarian insufficiency, with a normal karyotype and without *FMR1* gene permutation, and from a Control Group, who had menopause after 50 years of age. Frequencies were compared using Fisher's exact test.

**Results:**

A total of 100 samples from the Case Group and 100 samples from the Control Group were selected. The variant was detected in heterozygosity in a Case Group sample but was not identified in the Control Group.

**Discussion:**

This variant was first described in a consanguineous Arab family. This variant is classified as pathogenic and has a prevalence of 1% in women with primary ovarian insufficiency. This study is a pioneering investigation of this variant in Brazilian women.

**Conclusion:**

These findings suggest that the rs138761187 variant of the *MCM8* gene is rare in Brazilian women.

## In Brief

This cross-sectional, case-control study demonstrated that the *MCM8* gene might be an autosomal gene implicated in premature ovarian insufficiency. We evaluated the frequency of the NG_042869.1:g.40270G>A (rs138761187) variant in *MCM8* in Brazilian women with premature ovarian insufficiency and in those with normal menopause age. This study is a pioneering investigation of the rs138761187 variant of *MCM8* in women of Brazilian origin.

## Highlights

■ The MCM8 gene is involved in homologous recombination, acting in the ovarian follicles during meiosis II.
■ To the best of our knowledge, this is the first study to report the presence of this variant in a Brazilian woman.
■ The rs138761187 variant had a low incidence; further studies with a larger sample size or focusing on variants at other gene positions may provide insight into the genetic causes of premature ovarian insufficiency.

## Graphical abstract

**Figure:**
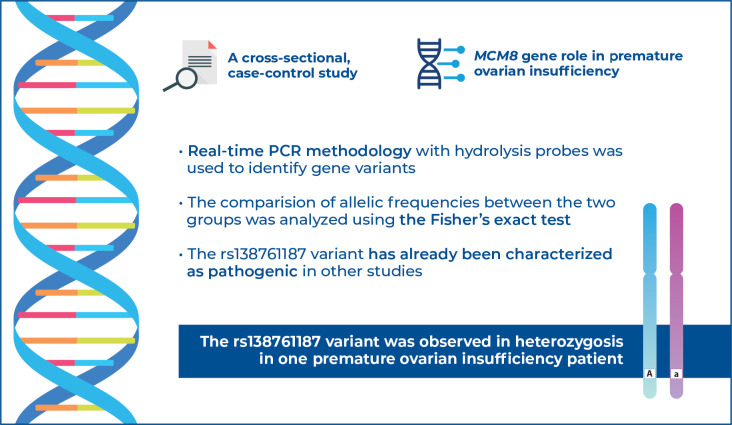

## INTRODUCTION

The median age at menopause in women is approximately 50 years.^(1)^ However, premature ovarian insufficiency (POI), also known as early menopause, occurs when the ovarian reserve is depleted before the age of 40 years and is observed in approximately 1% of women in this age group. It is characterized by amenorrhea, infertility, hypoestrogenism, and elevated serum follicle-stimulating hormone (FSH) levels.^(2)^ Although there is no diagnostic accuracy for POI, the Guideline Development Group of the World Health Organization recommends the following diagnostic criteria: oligo/amenorrhea for at least 4 months, associated with FSH values above 25 IU/L, measured on two occasions with an interval greater than 4 weeks. The etiology of POI is associated with autoimmune disorders, infections, enzyme deficiencies, hormonal signaling defects, iatrogenic exposure, chromosomal abnormalities, and alterations in genes, such as *FMR1* and *FSHR*. Furthermore, 10%-15% of cases have an affected first-degree relative, indicating a significant genetic etiology.^(3)^

The *MCM8* (minichromosome maintenance 8) gene an autosomal gene that has recently been implicated as a cause of POI. It is located on chromosome 20 and is a member of the MCM (*MCM2-9)* gene family.^(4)^ *MCM8* is expressed during prophase I, when double-stranded DNA breaks occur. These breaks can occur when the replicative helicase *MCM2-7* encounters a blockage and disengages from the DNA. The cells recruit the *MCM8-9* complex as a backup helicase, which continues to synthesize DNA on behalf of *MCM2-7*.^(5)^ When the *MCM8-9* helicase fails, the backup system does not function, leading to an accumulation of double-stranded DNA breaks and eventual cell death. Oocytes in the human ovary are more prone to this process as they rest precisely in prophase I, which is the period of meiosis when *MCM8* is expressed. Thus, variants of this gene can affect the fertility and lifespan of women.

In addition, it is worth noting that in gametogenesis, double-stranded DNA breaks are essential for the exchange of maternal and paternal information. *MCM8-9* helicase is the main repair mechanism for these ruptures.^(6)^ A previous study revealed that *MCM8* and *MCM9* deficiency impairs homologous recombination (HR) in mice. Malformed primary follicles, which were incapable of further development, were found in the ovaries of these mice. This is because a defective HR leads to an early block in follicular development and subsequent follicular deficiency during embryogenesis or early life. This study also demonstrated that female *MCM8* -/- mice were sterile, with an early loss of germ cells.^(6)^

Pathogenic variants of the *MCM8* gene have been described in women diagnosed with POI. In a Tunisian family with a high degree of consanguinity, an *MCM8* gene variant that causes a deficiency in the repair of chromosomal breakage, which can cause ovarian failure, was detected.^(7)^ In Saudi Arabia, the NG_042869.1:g.40270G>A variant was identified in three sisters with hypergonadotropic primary amenorrhea.^(8)^ In 2015, full-exome sequencing identified two new homozygous mutations in *MCM8*, one at the splicing site (c.1954-1G>A) and the other at the frameshift site (c.1469-1470 insTA)*.* Both mutations were not detected in Control Group. Homozygous individuals with these variants showed significantly lower *MCM8* gene function. Chromosomal breakage following exposure to mitomycin C significantly increased in cells from individuals homozygous for c.1954-1G>A and c.1469-1470 insTA.^(9)^

Therefore, dysfunction of *MCM8* has been shown to lead to impaired repair of double-stranded DNA breakage, resulting in germ cell depletion and a dysgenetic ovary, which characterize the POI phenotype.^(6-9)^ However, the functional significance of *MCM8* gene variants, as well as its frequency in different ethnic groups and its involvement in POI pathogenesis, remains unclear.

Therefore, the present study aimed to examine the frequency of a new variant of the new pathway *MCM8/MCM9* in Brazilian women with POI.

## OBJECTIVE

To evaluate the frequency of the NG_042869.1:g.40270G>A (rs138761187) variant in the *MCM8* gene in Brazilian women with premature ovarian insufficiency and in those with normal menopausal age.

## METHODS

This was a cross-sectional, case-control study performed using 100 DNA samples obtained from women with POI and 100 DNA samples from a Control Group composed of fertile women.

The NG_042869.1:g.40270G>A (rs138761187) variant of *MCM8* was analyzed in participants diagnosed with POI from the *Instituto Ideia Fértil* database and controls.

### Casuistry

DNA samples stored in a biobank that were obtained from women at the *Instituto de Saúde Reprodutiva Ideia Fértil* (Santo André, SP, Brazil) were evaluated. The POI Case Group included patients who had amenorrhea for more than 4 months, associated with serum estradiol levels below 50pmol/L and FSH levels above 25mIU/L, dosed twice, with a minimum interval of 1 month. The Control Group included women with at least one pregnancy and parity, who entered menopause at >50 years of age. The exclusion criteria were individuals who underwent chemotherapy or radiotherapy, those who underwent uni-or bilateral gonadectomy, and the presence of chromosomal alterations, gene premutation *FMR1*, thyroid and/or glucose metabolism abnormalities, and/or autoimmune diseases. The samples with poor DNA quality or failed amplification were also excluded.

A free and informed consent form was prepared in two copies: one for the researcher and the other for the participant. The document was completed by all study participants and is attached to this article.

### Collection of biological samples

DNA samples were obtained from the Genetics Laboratory biobank, which belongs to the *Instituto de Saúde Reprodutiva Ideia Fértil* (CONEP Registration B-061 - Process #25000.091276/2016-95). DNA was extracted from peripheral blood leukocytes using a salt-out method (Lahiri and Numberg, 1991).

The samples were quantified using NanoDrop (Thermo Fisher Scientific, USA) and diluted to 50ng/mL. This study was approved by the Research Ethics Committee of the *Centro Universitário FMABC* (CAAE: 38528620.9.0000.0082; #4.371.586).

### Real-time PCR

Gene variants were identified via real-time polymerase chain reaction (qPCR) using hydrolysis probes (TaqMan; Thermo Fisher Scientific) according to the manufacturer's instructions. The TaqMan assay (4351379) described in the literature was chosen to cover the rs138761187 variant of *MCM8*.

qPCR was used to amplify the DNA. For this, mixtures consisting of 6.25*μ*L of PCR master mix, 0.25*μ*L of TaqMan assay, and 4.00*μ*L of purified water were prepared, multiplied by the total number of reactions, including one for the negative control. Negative controls were individuals without mutations. No positive controls were used in this study. The mixture was homogenized and distributed in the determined number of 2*μ*L tubes of DNA diluted at 50ng/*μ*L was added to the corresponding tube, resulting in a total volume of 12.5*μ*L per tube. The tubes were placed in a thermocycler. The amplification of *MCM8* was performed for 45 cycles, and the results were generated by simple amplification.

### Statistical analysis

Allelic frequencies between the two groups were compared using Fisher's exact test. Statistical significance was set at p<0.05.

## RESULTS

We analyzed 100 samples from patients diagnosed with POI at *Instituto Ideia Fértil* (mean age=52.09, SD=6.05). The rs138761187 variant was detected in one heterozygous sample (Table 1). Fisher's exact test was performed, which resulted in a p=1.

**Table 1:** Genotype distribution of the MCM8 rs138761187 variant in patients with premature ovarian insufficiency and in controls

	Genotypes			Alleles		
	GG	GA	AA	G	A	p
Patients with POI	99	1	0	199	1	
Controls	100	0	0	200	0	
Total	199	1	0	399	1	1.005

POI: primary ovarian insufficiency.

Table 1 presents the genotype distribution of the *MCM8* rs138761187 variant. The GG genotype refers to homozygous samples, and the GA genotype refers to heterozygous samples. The rs138761187 variant was detected in one of the GA samples from a patient with POI.

In terms of the clinical findings of patients with POI, the mean values were as follows: current age, 38.13; age at the last menstrual period, 33.84; and serum FSH value, 51.93.

## DISCUSSION

**Figure 1:**
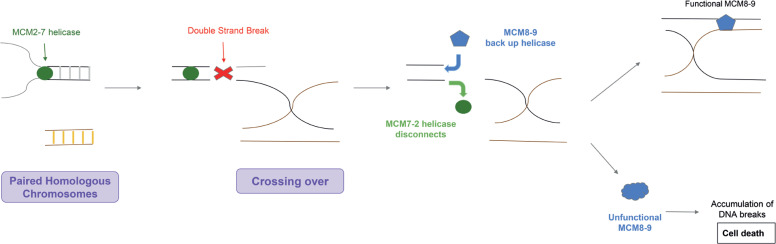
Summary of homologous recombination

The *MCM8* and *MCM9* genes are the last genes in their families to be described; thus, their functions are only partially known. Through dimerization with *MCM9*, the *MCM8-9* complex forms a repair helicase, replacing the replicative helicase *MCM2-7*, when a double-stranded DNA break occurs, as shown in figure 1. The *MCM8-9* helicase functions during prophase I, which explains the studies relating the *MCM8* gene to premature ovarian failure.

MCM2-7 helicase disengages from the chromosome upon encountering a double-stranded break. MCM8-9 then attaches to chromosomes as a backup helicase. If MCM8-9 does not function, double-stranded DNA breaks accumulate, leading to cell death.

**Figure 2:**
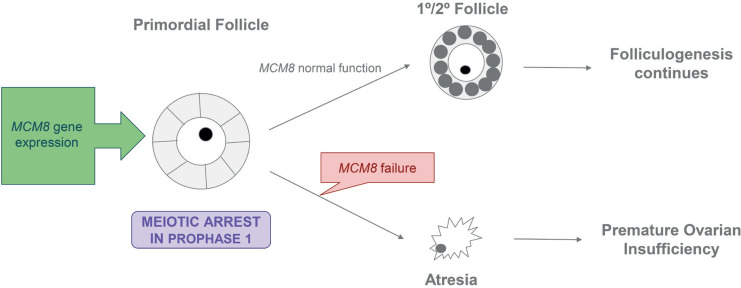
Role of the *MCM8* gene in folliculogenesis

Oocytes in the human ovary are arrested in prophase I and resume meiosis just before ovulation; therefore, the *MCM8-9* complex may be required to repair double-stranded DNA breaks during the follicular or ovulatory phase of the menstrual cycle. A Finnish Study evaluated the expression of *MCM8-9* in women with POI and a Control Group^(8)^ reported an increased expression of *MCM8* during the follicular phase compared with the ovulatory and luteal phases. This finding indicates that *MCM8* plays a major role in follicular recruitment and development. Therefore, *MCM8* gene variations can cause a follicular development disorder, resulting in a lack or inefficiency of its transcript. A study in mice showed that *MCM8* -/- ovaries contain malformed primary follicles that cannot develop further.^(6)^ In humans, several variants related to POI have been described, as shown in figure 2. Desai et al. sequenced the *MCM8* gene in 155 women with POI and identified 10 nonsynonymous variants in 18 women, of which 4 were benign and 2 were significantly associated with POI.^(2)^ All the identified variants were heterozygous.^(2)^

During folliculogenesis, the primordial follicle remains in meiotic arrest during prophase I. The *MCM8* gene acts precisely during this phase, allowing folliculogenesis to proceed. In cases of *MCM8* failure, folliculogenesis is interrupted, leading to atresia of the primordium, and ultimately, primary ovarian insufficiency.

The gene variant NG_042869.1:g.40270G>A (rs138761187) investigated in this study has been previously detected in two brothers from a family of Arab origin in a study published in April 2015.^(9)^ The eldest sister was completely prepubertal with primary amenorrhea at the age of 15 years and a karyotype of 46,XX. In addition, her gonadotropin levels were elevated, which was consistent with POI. The brother had elevated gonadotropin levels, both basal- and GnRH-induced, and azoospermia, consistent with primary testicular failure. His karyotype was 46,XX.^(9)^

This novel variant causes the c.1954-1G>A substitution, resulting in the lack of normal *MCM8* and production of three aberrant *MCM8* transcripts, with a significant reduction in the amount of mRNA transcribed from *MCM8.*^(9)^ Therefore, the hypothesis of the present study is that the rs138761187 variant would be more frequently detected in participants of the POI Group than in controls.

The findings of this study revealed the presence of the variant, which was previously described in primary homozygosity, in heterozygosity in a sample of the POI Group. This may be due to the fact that our study covers the general population whereas other studies investigating this variant rely on highly consanguineous families, making homozygosity more likely.

Our study is a pioneering investigation of the rs138761187 variant of *MCM8* in women of Brazilian origin. This variant has a low incidence worldwide; it has been reported in ClinVar in 1 / 8,599 (0.012%) European American exomes and 0 / 4,066 African American exomes on the Exome Variant Server as well as in 1 / 4,545 (0.022%) genomes in the 1000 Genomes Project database.^(10)^

This is the first study to demonstrate the presence of this variant in a Brazilian woman. In addition, this variant was not detected in participants who experienced menopause after 50 years of age, supporting the hypothesis that an alteration in the *MCM8* gene may relate to the POI phenotype.

A strong feature of the present study was the proper selection of the Case Group, excluding known causes of POI such as karyotype alterations and gene premutation. In addition, only women who were fertile and menopausal after 40 years of age were included in the Control Group. However, only one gene variant was investigated. Variants at other gene positions may be present in the sample at a frequency different from that of the observed variant. Furthermore, study sampling was based on convenience and accessibility to the *Instituto de Saúde Reprodutiva Ideia Fértil*.

Therefore, the present study is a pioneering investigation of the rs138761187 variant of *MCM8* in Brazilian women diagnosed with POI. In summary, our results provide an important contribution to the search for genetic causes of POI and the relevance of *MCM8* gene alterations in the development of this condition.

## CONCLUSION

This study demonstrated a low incidence of the rs138761187 variant of *MCM8*. However, these results do not exclude the possibility that variants of this gene are involved in the development of the condition.

Considering the main role of *MCM8* in the development and maintenance of gonads, our findings may contribute to elucidating the role of this variant in women with primary ovarian insufficiency.
